# Impact of geographical, meteorological, demographic, and economic indicators on the trend of COVID-19: A global evidence from 202 affected countries

**DOI:** 10.1016/j.heliyon.2023.e19365

**Published:** 2023-08-28

**Authors:** R.M. Ammar Zahid, Qamar Ali, Adil Saleem, Judit Sági

**Affiliations:** a School of Accounting, Yunnan Technology and Business University, Yunnan, PR China; b Department of Economics, Virtual University of Pakistan, Faisalabad Campus 38000, Pakistan; c Doctoral School of Economics and Regional Studies, Hungarian University of Agriculture and Life Sciences, H-2100 Gödöllő, Hungary; d Faculty of Finance and Accountancy, Budapest Business University — University of Applied Sciences, H-1149 Budapest, Hungary

**Keywords:** COVID-19, Environment, Geography, Generalized Poisson regression, Income, Population density

## Abstract

**Research problem:**

Public health and the economy face immense problems because of pathogens in history globally. The outbreak of novel SARS-CoV-2 emerged in the form of coronavirus (COVID-19), which affected global health and the economy in almost all countries of the world.

**Study design:**

The objective of this research is to examine the trend of COVID-19, deaths, and transmission rates in 202 affected countries. The virus-affected countries were grouped according to their continent, meteorological indicators, demography, and income. This is quantitative research in which we have applied the Poisson regression method to assess how temperature, precipitation, population density, and income level impact COVID-19 cases and fatalities. This has been done by using a semi-parametric and additive polynomial model.

**Findings:**

The trend analysis depicts that COVID-19 cases per million were comparatively higher for two groups of countries i.e., (a) average temperature below 7.5 °C and (b) average temperature between 7.5 °C and 15 °C, up to the 729th day of the outbreak. However, COVID-19 cases per million were comparatively low in the countries having an average temperature between 22.5 °C and 30 °C. The day-wise trend was comparatively higher for the countries having average precipitation between (a) 1 mm and 750 mm and (b) 750 mm and 1500 mm up to the 729th day of the outbreak. The day-wise trend was comparatively higher for the countries having more than 1000 people per sq. km. Discussing the COVID-19 cases per million, the day-wise trend was higher for the HICs, followed by UMICs, LMICs, and LIC.

**Conclusion:**

The study highlights the need for targeted interventions and responses based on the specific circumstances and factors affecting each country, including their geographical location, temperature, precipitation levels, population density, and per capita income.

## 1 Introduction and background

The emergence of a novel server acute respiratory syndrome named coronavirus 2 (SARS-CoV-2) poses serious considerations to public health and the global economy [1]. The virus was initiated in December 2019, in China. Its main symptoms are fever, cough, fatigue, sore throat, loss of taste or smell, and shortness of breath. Its transmission is observed through droplets or direct interaction between human beings [2]. The clinical symptoms of COVID-19 showed similarity with the symptoms of MERD and SARS [3]. It is declared a pandemic by the World Health Organization (WHO). More than 200 countries were affected due to COVID-19, accounting for 765,222,932 confirmed cases [4]. Due to the non-availability of proper treatment, the government focused to stop the spread by imposing lockdowns and quarantines [5]. The inhabitants have been guided to maintain social distancing, stay in their homes, wash their hands, and avoid gatherings to break the viral spread [6].

The COVID-19 spread might influence by political, social, geographical, and climatic factors [7]. Generally, it is believed that weather variables play a significant role in infectious diseases like Spanish inﬂuenza, SARS-COV, and MERS-COV [8]. In a study, Dalziel et al. [9] mentioned that virus spread is linked to factors like temperature, population density, and humidity. It is required to explore the effect of environmental, demographical, meteorological, and pollution variables on COVID-19 transmission [10].

The weather-related indicators affect COVID-19 spread in three channels. First, the virus and its stability are linked with ambient conditions like humidity and temperature–––weak stability at high temperature (37 °C) and high stability at low temperature (4 °C). Similarly, virus destabilization was observed due to humidity as it affects the protective lipid membrane. Second, meteorological indicators (i.e., humidity, wind speed, and air temperature) can affect the stability of potential carriers (i.e., air and contaminated surfaces). Third, meteorological indicators increase the vulnerability of human beings to adverse environments. Meteorological conditions may influence human activity (i.e., contact rates, travel, and immune function) [11].

Various studies indicate that weather conditions were responsible for 18% variation in the doubling time, while 82% variation is due to other demographic features [12]. Environmental conditions are vital in the initiation and dynamic behavior of many infectious viruses [13]. The ongoing pandemic was observed frequently in low-temperature regions (3–17 °C average annually). About less than 6% of COVID-19 cases were found in countries having an average temperature above 18 °C. In China, scientists observed the link between COVID-19 and humidity, temperature; the virus spread was less in warm and humid and areas. In Finland and Spain, researchers observed that 95% of global infectious diseases occur in the temperature range (2°C–10 °C), and dry weather. However, the COVID-19 pandemic was also observed in hot and humid regions like Singapore, Malaysia, and Indonesia [14].

Moreover, historical data reveals accelerated diffusion of epidemics during economic booms due to an increase in traveling and interpersonal contacts. Similarly, COVID-19 diffusion may influence due to economic indicators (i.e., employment, GDP per capita) [10]. Economic development can facilitate virus transmission due to modern transport systems, high population density, and traveling for vacation and business [15]. It is a common understanding that its transmission is high in populated areas [16]. However, the literature is less about the relationship between population density and COVID-19. The dynamics between COVID-19 and density are important for medical resources and planning [17]. The average family size is more in low-income countries which may increase COVID-19 spread. The closure and containment policies were more beneficial in developed economies than in low-income countries [18].

Keeping in view the current epidemiological dynamics of COVID-19, it is essential to understand the association between geographical factors and COVID-19 transmission to make the best decision to control and prevent the COVID-19 pandemic [14]. In addition, demographic and socioeconomic variables may influence COVID-19 transmission, however, the literature is limited on this topic [19]. Moreover, understanding the factors that influence the viral spread is important for several reasons (a) provision of appropriate scientific responses to complex mechanisms of COVID-19 transmission, (b) refinement of epidemiologic modeling of diffusion, and (c) support suitable policies of emergency management to tackle this threat [10].

Therefore, this study answers five research questions: First, what is the link between temperature and COVID-19? Second, what is the link between weather indicators and COVID-19? Third, what is the link between demographic variables and COVID-19? Fourth, what is the link between economic variables and COVID-19? And Fifth, Is there heterogeneity across different continents (i.e., Asia, Europe, Africa, North America, South America, and Australia & Oceania) in terms of COVID-19 cases?

These research questions have been addressed through the following specific objectives:

1. To study the impact of temperature and precipitation on COVID-19 cases.
2. To study the impact of population density and income on COVID-19 cases.

This research offers a significant addition to the literature in four ways. First, it examines the heterogeneity of daily new cases in different continents. It is helpful for policymakers to make a comprehensive policy to control COVID-19 transmission across different regions. Second, it examines the impact of two climate or meteorological indicators (i.e., annual average temperature and annual precipitation) on COVID-19 transmission. It is helpful for policymakers to consider meteorological indicators in policymaking to combat the pandemics like COVID-19. Third, it examines the impact of demographic (i.e., population density) and economic (i.e., per capita income) variables on COVID-19 transmission. It is helpful for policymakers to consider demographic and socio-economic indicators in health policy to tackle future outbreaks. Fourth, this study first time uses a large panel (i.e., 202 countries) to investigate the dynamic of COVID-19 cases across the globe.

## 2 Literature review

Epidemiological studies show the nexus between the meteorological state and COVID-19 [20]. Some studies [21,22] explained the link between climate change and the emergence of various infectious viruses. The literature revealed that weather dryness or coldness is responsible for the spread of influenza. The spread of SARS reduced in the hot season and ultimately ended in July 2003. Similarly, the COVID-19 pandemic was predominantly found in low-temperature regions [7]. Meteorological indicators (i.e., humidity, average temperature, and wind speed) inversely affected the COVID-19 cases [11]. The literature can be divided into four strands (a) temperature and COVID-19, (b) weather/meteorological indicators and COVID-19, (c) demographic indicators and COVID-19, and (d) economic variables and COVID-19.

The first strand indicates the impact of temperature on COVID-19 spread. Some studies reported that temperature leads to a reduction in COVID-19 transmission [7,13,23] while some studies reported a positive link between temperature and COVID-19 [24,25]. The COVID-19 incidence dropped by 0.86% for a 1 °C rise in the minimum temperature [26]. The authors, Xie and Zhu [2] stated that transmission of COVID-19 was influenced by temperature. In another study [20], the authors showed that the 1 °C increase in temperature resulted in a reduction of 3.08% and 1.19% in new cases and new deaths, respectively. Prata et al. [27] observed that variation in average annual temperature could impact the transmission of COVID-19. Jahangiri et al. [28] described that temperature and population size may influence the COVID-19 pandemic. A nonlinear reduction in COVID-19 cases was observed due to an increase in mean temperature in winter [19]. The second strand indicates the impact of weather/meteorological indicators on COVID-19. Most studies reveal an opposite connection between COVID-19 spread and humidity [20,29]. A 1% rise in relative humidity decreased new cases and deaths to 0.85% and 0.51%, respectively. The correlation between weather indicators and COVID-19 was studied by Tosepu et al. [3] in Jakarta and Indonesia. Meteorological factors also influence COVID-19 spread [30]. Bashir et al. [31] observed clear linkages between climate descriptors and COVID-19 in New York and the United States. A nonlinear increase in cases was observed due to an increase in absolute humidity [19]. The third strand indicates the impact of demographic factors on COVID-19. In Turkey, Şahin [32] showed an association between the population, temperature, dew point, wind speed, and COVID-19. Bhadra et al. [16] showed a moderate link between population density and COVID-19 cases. Selcuk et al. [33] reported a positive link between COVID-19 cases and population density in Turkey. Similarly, Sharif and Dey [34] described a strong positive link between COVID-19 cases and population density in Bangladesh. However, Sun et al. [35] mentioned that population density is not a vital determinant of COVID-19 transmission under strict lockdown. The fourth strand indicates the impact of economic indicators on COVID-19. Baena-Díez et al. [36] revealed a higher probability of COVID-19 transmission in the presence of lower income in Spain. Similarly, low-income districts had greater COVID-19 transmission per 10,000 while high-income districts had low COVID-19 transmission.

## 3 Methods

This study used COVID-19 data from 202 countries from January 03, 2020, to December 31, 2021. This study used data about daily cases and deaths, collected from the WHO COVID-19 Dashboard [4]. A total of 202 countries was investigated by dividing the countries according to their continent, temperature, precipitation, population density, and income level. Thus, this is an ecological study, and data on COVID-19 and ecological variables were obtained from secondary sources. According to Tsinda and Mmbando [37], there exists heterogeneity across continents in terms of COVID-19 burden. Therefore, we performed separate analysis for each continent to explore the spatial heterogeneity of the impact of ecological variables on COVID-19. The data about average annual temperature and average annual precipitation was obtained from the weather base [38]. The data about population density and per capita income were obtained from World Development Indicators [39]. The transmission rate describes the speed of COVID-19 transmission in a country. This study also analyses the trend of infection rate in 202 countries, using the formula [14]:

(1) $$Transmission\;Rate\;\left(TR\right)=\frac{Number\;of\;Infected\;Persons}{Days\;of\;Infection\;\left(Outbreak\right)}$$

This study explores the day-wise trend of COVID-19 confirmed cases, deaths, and infection rates in 202 affected countries. This study explored the trend of these variables by categorizing 202 countries based on continent/geography (Appendix 1), average annual temperature (°C) (Appendix 2), average annual precipitation (mm) (Appendix 3), population density (people per sq. km) (Appendix 4), and average annual income per capita (USD) (Appendix 5).

### 3.1 Poisson regression analysis

Poisson regression is a statistical method that is specifically designed to predict outcomes that are counted, such as COVID-19 cases and fatalities. Poisson regression is a technique that is used to predict count outcomes, occurring in a given space or time [40]. Oftentimes, the observed counts reflect low-frequency events [41]. As the dependent variable of the study is the number of COVID-19 cases and Fatalities (count variable), therefore we used the Poisson regression model. Unlike ordinary least squares (OLS) regression, Poisson regression doesn't assume that the residuals (the differences between the predicted and actual values) follow a normal distribution with consistent variance [41]. Thus, we have used Poisson regression as our modeling technique.

Specifically, following the study [42], we have applied the Poisson regression method to assess how temperature, precipitation, population density, and income level impact COVID-19 cases and fatalities. This has been done by using a semi-parametric and additive polynomial model, as described by Prata et al. [27] and described in the following equation:

(2) $$\log\;{TC}_{t}=\beta_{0}+\beta_{1}X_{t}^{3}+\beta_{2}X_{t}^{2}+\beta_{3}X+s\left({Temp}_{t}\right)+s\left({Prec}_{t}\right)+s\left({PD}_{t}\right)+s\left({Income}_{t}\right)+\varepsilon_{t}$$

where TC is the total number of COVID-19 instances across the world and continents on day t, $\beta_{0}$ is the intercept, $\beta_{1-3}$ represents the parameter of x, while x represents the linear variable count days on day t, which indicates the number of days from the initial epidemic. ${Temp}_{t}$ denotes average daily temperature (°C), ${Prec}_{t}$ shows average daily precipitation (mm), ${PD}_{t}$ represent the population density, and ${Income}_{t}$ represents average income levels. Using equation s (−), the explanatory variables were corrected for confounding. Ma, et al. [30] discovered that the smoothing spline function s (−) can control the impact of confounding variables. The empirical study was conducted using STATA 15.

Li et al. [43] also investigated the relationship between total cases and meteorological factors using the Poisson model. Poisson regression is a frequently used classical model based on classical assumptions. Further, it is assumed that the dependent variable has a Poisson distribution, with values of 0, 1, 2, 3, … n. It describes the distribution of $y_{i}$ or the anticipated value of $y_{i}$, as given below [44]:

(3) $$E\left\{{TC}_{i}|x_{j}\right\}=\exp\left\{x_{j}^{T}\beta \right\}$$

The models based on count data assumed that the count variable ${TC}_{i}$ for a given $x_{i}$ follows a Poisson distribution.

(4) $$P\left\{{TC}_{i}=TC|x_{j}\right\}=\frac{\exp\left\{-\lambda_{i}\right\}\lambda_{j}^{TC}}{TC!},TC=0,1,2,3,\ldots$$

where TC! indicates TC's factorial. Substituting the proper functional form for $\lambda_{i}$ yields expressions for the probabilities, which are then used to generate the log-likelihood function for this model, also known as the Poisson regression model. Count data with any sort of dispersion may be explained using the extended Poisson regression. It takes into account both positive and negative correlations between response variables [45,46]. The generalized Poisson regression is advantageous when over-dispersion ($Var\left({TC}_{i}\right)>E\left({TC}_{i}\right)$) and under-dispersion ($Var\left({TC}_{i}\right)<E\left({TC}_{i}\right)$) are present. The probability density function of ${TC}_{i}$ according to the generalized Poisson distribution is [47]:

(5) $$f_{i}\left({TC}_{j},\mu_{i},\alpha \right)=\left(\frac{\mu_{i}}{1+\alpha \mu_{i}}\right)\frac{{\left(1+\alpha {TC}_{i}\right)}^{{TC}_{i}-1}}{{TC}_{i}!}\exp\left[\frac{\mu_{i}\left(1+\alpha {TC}_{i}\right)}{1+\alpha \mu_{i}}\right]$$

where ${TC}_{i}=0,1,2,\ldots \ldots .\;and\;\mu_{i}=\mu_{i}\left(x_{i}\right)=\exp\left(x_{i}\beta \right)$, $x_{i}$ represents a (k-1)-dimensional vector of variables, such as driving behaviors, demographic characteristics, and medicine usage, and represents a (k-1)-dimensional vector of regression parameters. Further, it was assumed:

Mean: $E\left({TC}_{i}|x+i\right)=\mu_{i}$.

Variance: $V\left({TC}_{i}|x_{i}\right)=\mu_{i}{\left(1+\alpha \mu_{i}\right)}^{2}$.

Generalized Poisson regression is the extension of conventional Poisson regression [47]. The correlation between climatic factors and COVID-19 infection rate was also evaluated using extended Poisson regression.

## 4 Results and discussions

### 4.1 Descriptive statistics

Table 1 reports the descriptive statistics (mean, median, standard deviation, percentiles) of climatic indicators (temperature and precipitation), population density, income level, daily COVID-19 cases, COVID-19 transmission rate (TR), and COVID-19 fatalities in the world and on different continents. Continent Africa had a higher while continent Europe had a minimum mean temperature than others. The average daily precipitation in the world was 1064.52 mm. Continent Australia & Oceania had the highest while the continent Europe had the lowest precipitation. Europe is the most populated continent while North America is the least populated continent. During this pandemic, an average (median) of 47 covid new cases per day were recorded in the world. North America has the highest number of new cases per day with a median of 510 and Australia and Oceania have the lowest median of zero. The median of fatalities worldwide is zero while North America has the highest number of fatalities (10), followed by Asia (2) and North America (2).

**Table 1:** Descriptive statistics.

	New Cases	Total Cases	CasesPerM	Deaths	Total Deaths	TR	Temperature	Precipitation	Population Density	Income Level
Panel A: World										
**N**	147258	147258	147258	147259	147258	147258	147258	147258	147258	147258
**Mean**	1947.8	505268.4	22420.5	37.2	11337.2	997.6	18.9	1073.8	335.3	2.1
**Median**	47	13206	3404.6	0	195	35.9	21.5	957.7	89.4	2
**Std. Div**	10663.7	2591704	37287.3	178.9	50060.4	4685.9	7.7	666.5	1484.1	1.1
**Min**	0	0	0	0	0	0	−4.6	0	0.136	1
**P25**	0	620	291.7	0	8	2.5	12.5	573.8	35.8	1
**P75**	613	164993	28857.0	9	2978	394.2	25.7	1500.4	219.8	3
**Max**	474309	5.35E+07	300262.3	8786	819055	73435.2	28.5	2835	19196	4
**Panel B: Continent-wise (Median)**										
**Asia**	253	84290	3644.9	2	923	222.9	22.4	652.6	112.1	2
**Europe**	180	39013.5	23620.9	2	752	136.4	9.6	748.5	112.8	1
**Africa**	14	7717	876.5	0	123	20.3	23.8	1042.85	51.9	3
**South America**	5	2676	5297.7	0	33	6.3	25.5	1355.6	210.3	1
**North America**	510	122795	15017.6	10	3064	396.9	19.5	1059.1	19.7	2
**Australia & Oceania**	0	954	634.5	0	7	2.8	24.6	1980.2	33.7	1

### 4.2 The trend of COVID-19 confirmed cases in different continents

Table 2 shows the median of day-wise Covid-19 new cases in overall world, Asia, Europe, Africa, South America, North America, and Australia & Oceania. As of December 31, 2021, COVID-19 confirmed cases were reported in 45 Asian countries, 50 European countries, 52 African countries, 13 South American countries, 34 North American countries, and 8 Australian & Oceania countries. Due to limited space, the continent-wise comparison was made on the 15th, 30th, 50th, 60th, 90th, 100th, 120th, 150th, 180th, 200th, 210th, 240th, 250th, 270th, 300th, 330th, 350th, 360th, 390th, 400th, 420th, 450th, 480th, 500th, 510th, 540th, 550th, 570th, 600th, 630th, 650th, 660th, 690th, 700th, and 729th days of the outbreak. The day-wise median of confirmed COVID-19 new cases showed that the situation was different across time and continents. The median of new cases on the 90th day (3 months) of the COVID-19 outbreak was higher in Europe (63.5). Europe also showed the highest median of day-wise COVID-19 cases on the 100th day of the outbreak (55.5). The highest median of daily COVID-19 cases was 57 on the 120th day of the outbreak in Asia and South America. After that, South America showed a higher median of daily COVID-19 cases on 150th day (272), 180th day (410), 200th day (631), 210th day (914), 240th day (933), 250th day (615), and 270th day (696) of the COVID-19 outbreak. After that, Europe showed a higher median of daily COVID-19 cases on 300th day (920.5), 330th day (1377), 350th day (1416.5), and 360th day (501.5) of the COVID-19 outbreak. After that, South America showed higher median of daily COVID-19 cases on 390th day (666), 400th day (949), 420th day (1204), 450th day (1877), 480th day (2162), 500th day (2518), 510th day (2517), 540th day (2148), and 550th day (1206) of the COVID-19 outbreak. After that, Asia showed a higher median of daily COVID-19 cases on the 570th day (995), 600th day (1366), and 630th day (1144) of the COVID-19 outbreak. Again, Europe showed a higher median of daily COVID-19 cases on the 650th day (935), 660th day (1311), 690th day (816), 700th day (1905), and 729th day (2012.5) of the COVID-19 outbreak. This difference in the trend of COVID-19 may be due to several factors. However, this study empirically confirmed that geography (continent) is one of the possible factors behind the difference in COVID-19 spread. Some possible factors behind the difference may be temperature, precipitation, population density, mobility, health sector, demographic factors, and other related factors.

**Table 2:** Day-wise confirmed cases (Median).

Days	Asia	Europe	Africa	South America	North America	Australia & Oceania	World
15	0	0	0	0	0	0	0
30	0	0	0	0	0	0	0
50	0	0	0	0	0	0	0
60	0	0	0	0	0	0	0
90	21	**63.5**	1.5	11	0	1	9
100	45	**55.5**	2	17	1	0.5	11
120	**57**	34	8	**57**	0	0	10
150	41	15	20	**272**	0	0	19
180	202	25	12.5	**410**	0	0	16
200	211	61.5	17	**631**	1	0.5	29
210	274	84.5	27	**914**	2	0	59.5
240	175	116.5	10	**933**	20.5	21	68
250	137	97	25.5	**615**	11.5	1	51.5
270	298	114	11	**696**	11.5	0	42.5
300	527	**920.5**	8.5	515	7	0	90.5
330	555	**1377**	16.5	908	14	3.5	110.5
350	776	**1416.5**	24	1105	18	0.5	160.5
360	485	**501.5**	17	469	0	0	95.5
390	411	542	57.5	**666**	34	0.5	175
400	407	879.5	63.5	**949**	23	3	182.5
420	362	836	57	**1204**	18.5	2	190.5
450	510	1201	51	**1877**	11	2	190
480	953	360	32.5	**2162**	3	0	152.5
500	377	271.5	10	**2518**	4.5	2.5	118.5
510	840	336	11	**2517**	16.5	2	131
540	859	64.5	55	**2148**	10	1	101.5
550	1051	38.5	30	**1206**	2.5	0.5	56
570	**995**	119.5	61	919	29.5	1	135.5
600	**1366**	437	98.5	249	19	40.5	218.5
630	**1144**	813	71.5	411	89	34	259
650	714	**935**	27	412	64.5	76	138.5
660	528	**1311**	15	536	32.5	0.5	142.5
690	313	**816**	7.5	668	7	14	90
700	414	**1905**	24.5	649	15.5	9.5	137
729	499	**2012.5**	387	1697	257	15	517

### 4.3 The trend of COVID-19 according to temperature in the world

**Fig. 1:**
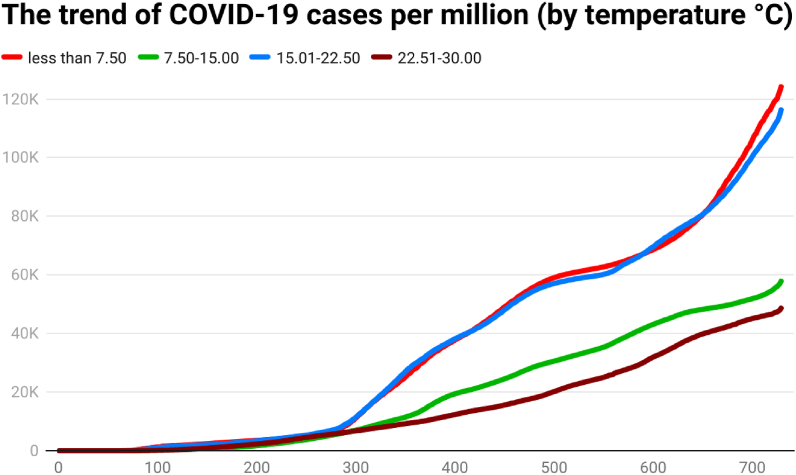
The trend of COVID-19 cases per million (by temperature °C).

**Fig. 2:**
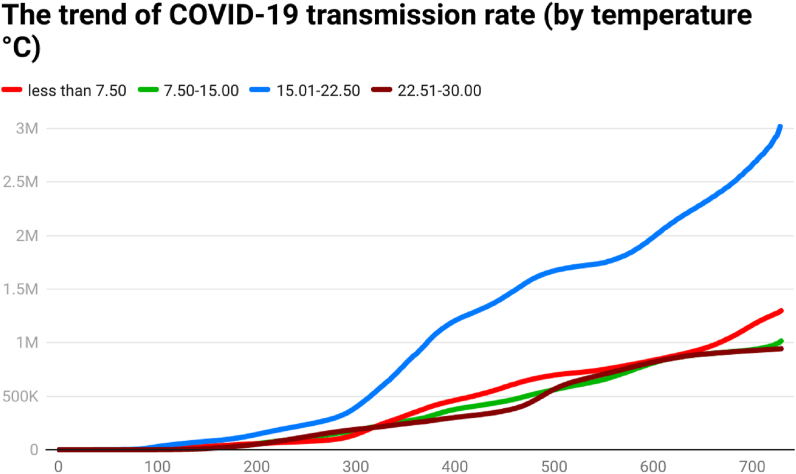
The trend of COVID-19 transmission rate (by temperature °C).

**Fig. 3:**
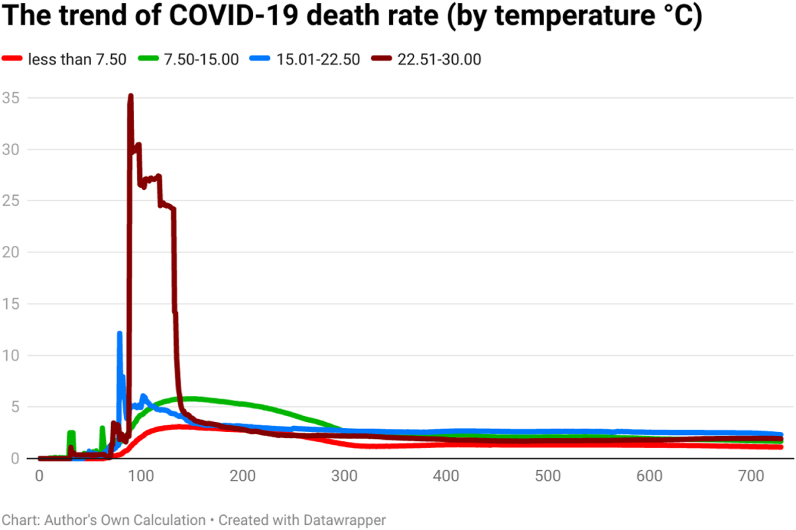
The trend of COVID-19 death rate (by temperature °C).

This study examines the day-wise trend of COVID-19 confirmed cases per million (Fig. 1), COVID-19 transmission rate (Fig. 2), and COVID-19 death rate (Fig. 3) by categorizing 202 countries into four panels based on average annual temperature. The four panels are (a) countries having an average annual temperature below 7.5 °C, (b) countries having an average annual temperature 7.5°C–15 °C, (c) countries having an average annual temperature 15.01°C–22.5 °C, and (d) countries having an average annual temperature 22.51°C–30 °C. Discussing the COVID-19 cases per million, the day-wise trend was comparatively higher for two groups of countries i.e., (a) average temperature below 7.5 °C and (b) average temperature between 7.5 °C and 15 °C, up to the 729th day of the outbreak. However, COVID-19 cases per million were comparatively low in the countries having an average temperature between 22.5 °C and 30 °C.

Another indicator, the COVID-19 transmission rate, also shows that the trend was also higher for the countries having a temperature between 15 °C and 22.5 °C. The transmission rate was less for the countries having a higher average temperature (22.5°C–30 °C). It seems that there might be a link between temperature and the spread of COVID-19.

The death rate of COVID-19 was comparatively higher for countries having a higher average temperature (22.5°C–30 °C). The COVID-19 death rate shows a huge difference across countries in the first year of the pandemic (i.e., approximately 350–400 days). It implies that the COVID-19 death rate had a direct association with average temperature. Contrary to this, the death rate of COVID-19 was comparatively low for countries having a lower average temperature (less than 7.50 °C) in the first year of the outbreak.

### 4.4 The trend of COVID-19 according to precipitation in the world

**Fig. 4:**
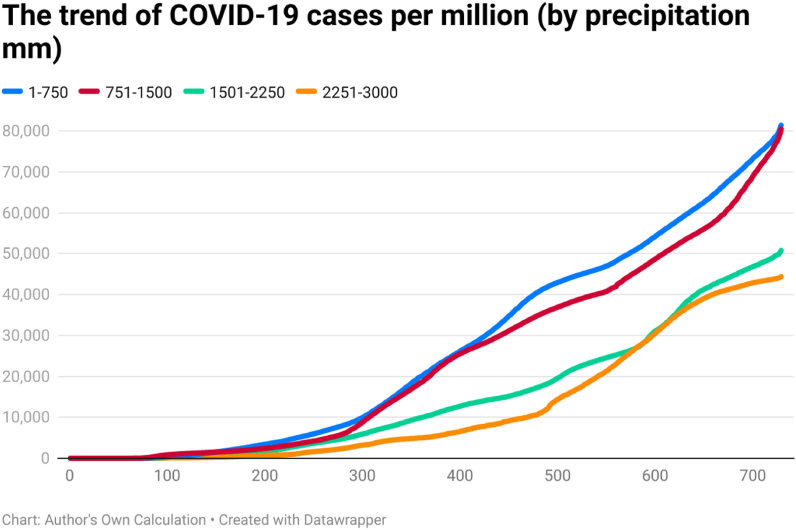
The trend of COVID-19 cases per million (by precipitation mm).

**Fig. 5:**
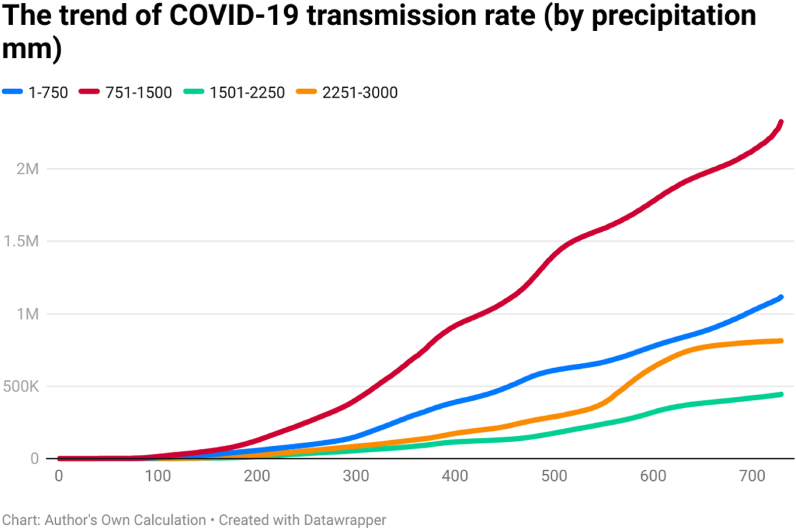
The trend of COVID-19 transmission rate (by precipitation mm).

**Fig. 6:**
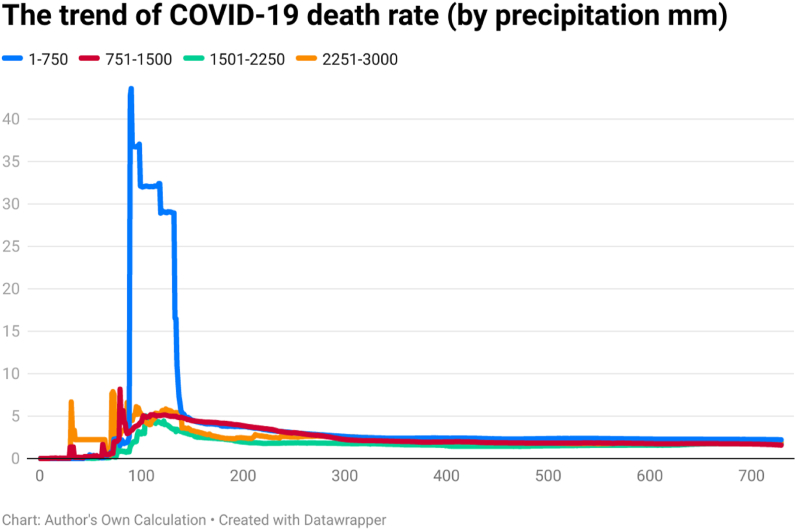
The trend of COVID-19 death rate (by precipitation mm).

This study examines the day-wise trend of COVID-19 confirmed cases per million (Fig. 4), COVID-19 transmission rate (Fig. 5), and COVID-19 death rate (Fig. 6) by categorizing 202 countries into four panels based on average annual precipitation. The four panels are (a) countries having precipitation between 1 mm and 750 mm, (b) countries having precipitation between 751 mm and 1500 mm, (c) countries having precipitation between 1501 mm and 2250 mm, and (d) countries having precipitation between 2251 mm and 3000 mm. Discussing the COVID-19 cases per million, the day-wise trend was comparatively higher for the countries having average precipitation between (a) 1 mm and 750 mm and (b) 750 mm and 1500 mm up to the 729th day of the outbreak. However, COVID-19 cases per million were comparatively low in countries having precipitation between 2251 mm and 3000 mm. It seems that there is an inverse association between precipitation and the spread of COVID-19 per million.

The COVID-19 transmission rate is also higher in countries having precipitation between 751 mm and 1500 mm. The transmission rate was less for the countries having precipitation between 1501 mm and 2250 mm. It seems that there is an association between precipitation and the spread of COVID-19. The trend of COVID-19 deaths rate was also higher in those countries having precipitation between 1 mm and 750 mm. The COVID-19 death rate was comparatively low for countries having precipitation between 1501 mm and 2250 mm. The COVID-19 death rate shows a huge difference across countries in the first year of the pandemic (i.e., approximately 300 days). However, the difference was less across four groups after 300 days of the outbreak.

### 4.5 The trend of COVID-19 according to population density in the world

**Fig. 7:**
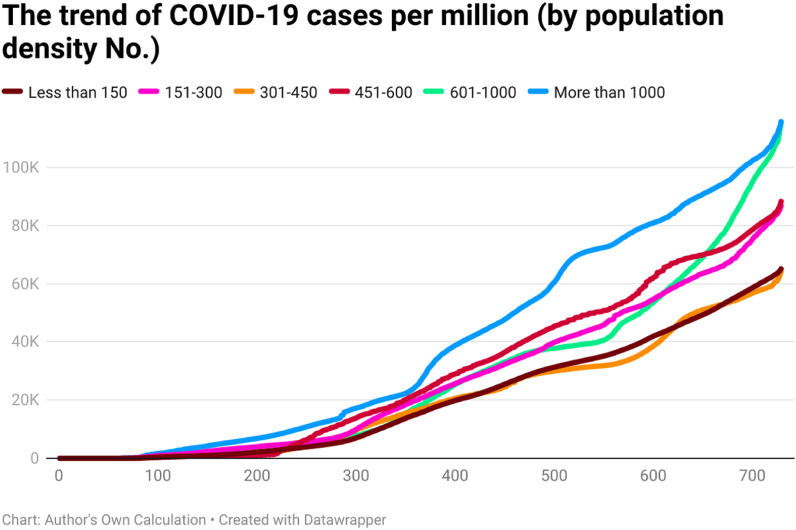
The trend of COVID-19 cases per million by population density (people per sq. km).

**Fig. 8:**
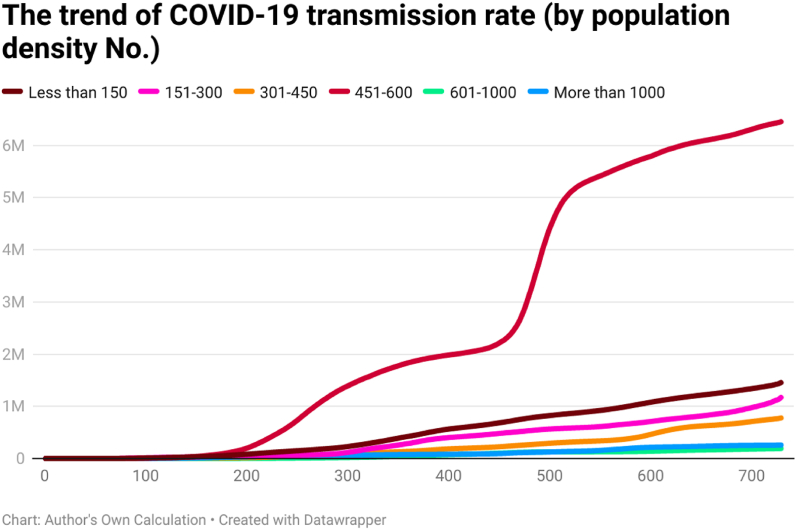
The trend of COVID-19 transmission rate by population density (people per sq. km).

**Fig. 9:**
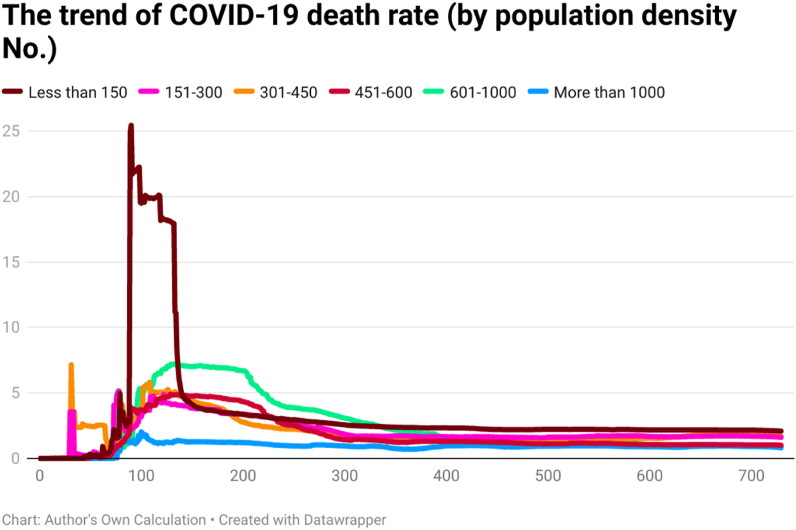
The trend of COVID-19 death rate by population density (people per sq. km).

This study examines the day-wise trend of COVID-19 cases per million (Fig. 7), COVID-19 transmission rate (Fig. 8), and COVID-19 death rate (Fig. 9) by categorizing the 202 countries into six panels based on population density. These panels are (a) countries with less than 150 people per sq. km, (b) countries having 151 to 300 people per sq. km, (c) countries having 301 to 450 people per sq. km, (d) countries having 451 to 600 people per sq. km, (e) countries having 601 to 1000 people per sq. km, and (f) countries having more than 1000 people per sq. km. Discussing the COVID-19 cases per million, the day-wise trend was comparatively higher for the countries having more than 1000 people per sq. km. However, COVID-19 cases per million were comparatively low in countries having population density (a) between 301 people per sq. km and 450 people per sq. km and (b) less than 150 people per sq. km. It seems that there is a direct association between population density and the spread of COVID-19 per million.

The COVID-19 transmission rate shows that the trend was higher for the countries having a population density between 451 people per sq. km and 600 people per sq. km. The transmission rate was less for the countries having population density between 601 people per sq. km and 1000 people per sq. km. The COVID-19 death rate shows that the trend was higher for countries having less than 150 people per sq. km. Contrary to this, the trend of COVID-19 death rate was comparatively less for countries having more population density (more than 1000 people per sq. km). It seems an inverse link between population density and the COVID-19 death rate.

### 4.6 The trend of COVID-19 according to per capita income in the world

**Fig. 10:**
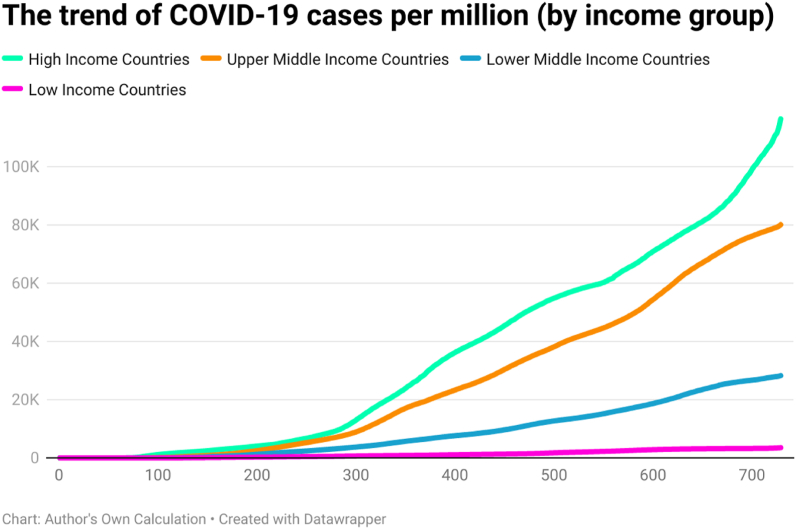
The trend of COVID-19 cases per million by per capita income (USD).

**Fig. 11:**
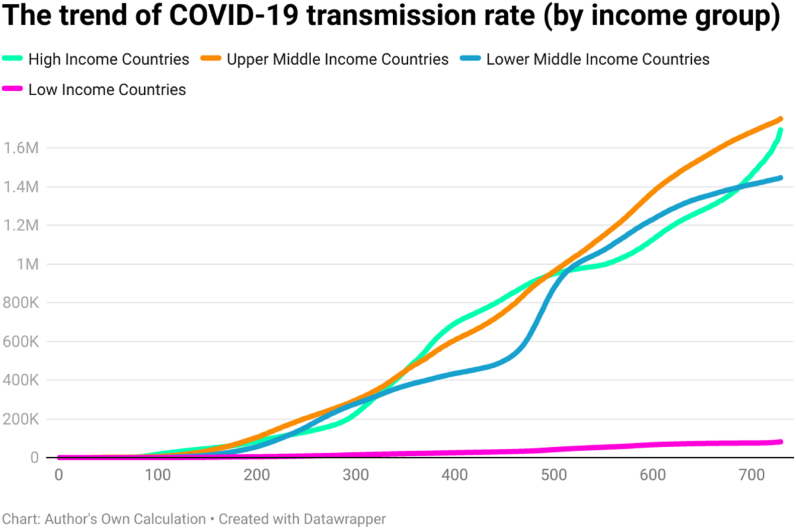
The trend of COVID-19 transmission rate by per capita income (USD).

**Fig. 12:**
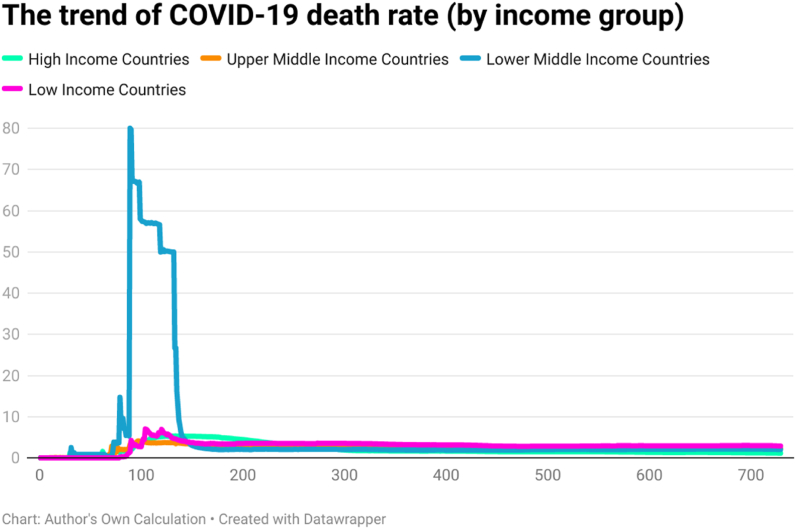
The trend of COVID-19 death rate by per capita income (USD).

This study examined the day-wise trend of COVID-19 cases per million (Fig. 10), COVID-19 transmission rate (Fig. 11), and COVID-19 death rate (Fig. 12) by categorizing 202 countries into four panels based on per capita income. These four panels are (a) high-income countries (HICs), (b) upper-middle-income countries (UMICs), (c) lower-middle-income countries (LMICs), and (d) low-income countries (LICs). Discussing the COVID-19 cases per million, the day-wise trend was higher for the HICs, followed by UMICs, LMICs, and LICs. It implies that COVID-19 confirmed cases had a direct association with income per capita. The COVID-19 confirmed cases were fewer in LICs, possibly due to low testing capability.

The trend of the COVID-19 transmission rate was also higher for the HICs, followed by UMICs, LMICs, and LICs. It is because high-income countries performed more tests for the detection of COVID-19. The trend of COVID-19 death rate was higher in LMICs. Compared to LMICs, the total number of COVID-19 deaths was less in other countries (HICs, UMICs, and LICs). However, the variation in death rate was less across all groups after 200 days of the COVID-19 outbreak approximately. Looking at the second year of the pandemic, it is clear that the death rate was comparatively low in HICs and comparatively high in LICs. All these trend lines show that there is a relationship between income level and COVID-19 intensity.

### 4.7 Correlation analysis

Table 3 Panel A to Panel G presents Pearson’s correlations between the key variables. It shows the pairwise correlations between Total COVID-19 new cases, total deaths, transmission rate, temperature, precipitation, population density, and income level of the overall world and continents. As expected, COVID-19 new cases, total deaths, and transmission rate are significantly positively correlated with each other in the overall world and all the continents. Correlations coefficients indicate the negative and significant association between the temperature, precipitation, population density, income level, and COVID-19 cases, deaths, and transmission rate (TR) in the overall world. The temperature has a significant positive correlation with Covid-19 cases, deaths, and transmission rates in Europe and North America. The COVID-19 spread is attributed to temperature as an environmental element [13]. The findings are in line with Şahin [32], who revealed that a decrease in Turkey's average temperature led to a rise in COVID-19 cases. Sobral et al. [48] also showed that a temperature rise led to a decrease in infections. Bi et al. [49] similarly found an inverse association between SARS transmission and temperature in Beijing and Hong Kong in previous research. In contrast, the findings are contradictory to previous country-level findings such as Jakarta, Indonesia [3], New York, US [31], and Pakistan [50]. The disparate outcomes between temperature and COVID-19 instances are because the influence of temperature on human health may vary among areas and nations [48,51]. Literature indicates that influenza and SARS-CoV viruses can only live under specified climatic circumstances, and their transmission rate is temperature-dependent [52,53]. Second, the negative and substantial association between average precipitation and COVID-19 cases, deaths, and TR worldwide and in all the continents (except Asia) suggests a decline in COVID-19 instances. It suggests that the increase in precipitation is associated with a decline in COVID-19 instances. Turkey has similarly observed a decline in COVID-19 instances owing to an increase in humidity [32]. Chan et al. [52] noted that increasing relative humidity reduces viral viability, supporting the conclusions of this study. In addition to these environmental elements, population [54], population density, medical treatment [26], people's endurance, social distance, and health facilities [31] may contribute to the spread or containment of COVID-19. This Pearson’s pairwise correlation analysis provides us with the initial evidence between the Total Cases, Total Deaths, TR, Temperature, Precipitation, Population Density, and Income Levels. However, further powerful tests are required to access the causality of the relationship so we applied the Poisson regression.

**Table 3:** Pearson's Pairwise correlations (selected variables).

Variables	Total Cases	Total Deaths	TR	Temperature	Precipitation	Population Density	Income Level
Panel A: World							
Total Cases							
Total Deaths	0.929*						
TR	0.972*	0.917*					
Temperature	−0.075*	−0.087*	−0.083*				
Precipitation	−0.039*	−0.054*	−0.045*	0.386*			
Population Density	−0.023*	−0.032*	−0.025*	0.011*	0.018*		
Income Level	−0.058*	−0.070*	−0.066*	0.379*	0.117*	−0.138*	
**Panel B: Asia**							
Total Cases							
Total Deaths	0.960*						
TR	0.977*	0.936*					
Temperature	−0.012*	−0.053*	−0.016*				
Precipitation	0.018*	0.023*	0.014*	0.408*			
Population Density	−0.027*	−0.041*	−0.028*	0.224*	0.249*		
Income Level	0.062*	0.075*	0.066*	−0.089*	0.097*	−0.250*	
**Panel C: Europe**							
Total Cases							
Total Deaths	0.939*						
TR	0.973*	0.961*					
Temperature	0.027*	0.051*	0.035*				
Precipitation	−0.111*	−0.121*	−0.124*	−0.142*			
Population Density	−0.075*	−0.078*	−0.083*	0.277*	−0.01		
Income Level	−0.055*	−0.060*	−0.063*	0.014*	−0.104*	−0.107*	
**Panel D: Africa**							
Total Cases							
Total Deaths	0.977*						
TR	0.963*	0.944*					
Temperature	−0.342*	−0.314*	−0.372*				
Precipitation	−0.142*	−0.150*	−0.155*	0.192*			
Population Density	−0.056*	−0.060*	−0.063*	−0.097*	0.206*		
Income Level	−0.215*	−0.205*	−0.238*	0.284*	0.102*	−0.006	
**Panel E: South America**							
Total Cases							
Total Deaths	0.948*						
TR	0.970*	0.940*					
Temperature	−0.253*	−0.273*	−0.279*				
Precipitation	−0.116*	−0.145*	−0.127*	0.270*			
Population Density	−0.132*	−0.163*	−0.145*	0.275*	−0.170*		
Income Level	−0.095*	−0.072*	−0.105*	0.141*	0.513*	−0.133*	
**Panel F: North America**							
Total Cases							
Total Deaths	0.966*						
TR	0.968*	0.934*					
Temperature	0.170*	0.133*	0.188*				
Precipitation	−0.143*	−0.206*	−0.163*	0.697*			
Population Density	0.086*	0.104*	0.096*	0.166*	−0.174*		
Income Level	0.078*	0.103*	0.086*	0.534*	0.136*	0.084*	
**Panel G: Australia & Oceania**							
Total Cases							
Total Deaths	0.888*						
TR	0.965*	0.925*					
Temperature	−0.150*	−0.232*	−0.215*				
Precipitation	−0.247*	−0.387*	−0.350*	0.729*			
Population Density	−0.141*	−0.182*	−0.174*	0.535*	0.362*		
Income Level	−0.041*	−0.093*	−0.082*	0.246*	0.697*	−0.271*	

***p < 0.01, **p < 0.05, *p < 0.1.

### 4.8 Generalized Poisson regression results

Using Poisson regression, Table 4, Table 5 illustrate the influence of average temperature, daily precipitation, population density, income level, and the number of days on the COVID-19 cases (cumulative) & deaths (cumulative) for the world (202 countries) and its continents (Asia, Europe, Africa, South America, North America, and Australia & Oceania), respectively. Model fitness tests are provided at the bottom of each table, for instance, the likelihood ratio (LR) chi-square test values are statistically significant in both tables showing that our model is fit. Similarly, the values of the pseudo-R-square (MacFadden’s pseudo-R-square) also represent that the variables explain the major relationship. Further, the ratio of the deviance and Pearson chi-square (goodness of fit) post estimation tests values are insignificant providing the evidence that our model is a good fit [41,55]. To explain the results of passion regression in a meaningful way we calculated the incidence rate ratio (IRR) “a measure that indicates the expected change in the incidence rate for each unit increase in the predictor variable”. An IRR greater than 1 suggests that an increase in the predictor score will result in a corresponding increase in the incidence rate, while an IRR less than 1 indicates a decrease in the incidence rate with an increase in the predictor score. Temperature and precipitation are considered to be key determinants in the propagation of coronaviruses from an environmental perspective [56]. Results in Table 4 show that the IRR for ‘temperature’ indicates that for each unit increase, the predicted incidence rate changes by a factor (0.991***, 0.996***, 0.993***, 0.978***, 0.990***, 0.993***) less than one for overall world and continents except the north American countries 1.032***, respectively (meaning that the incidence rate of both the new Covid-19 cases and deaths are decreasing). Similarly, Table 5 shows the IRR for ‘temperature’ indicates that for each unit increase, the predicted incidence rate changes by a factor (0.982***, 0.994***, 0.968***, and 0.948***) less than one for the overall world and continents except Europe, North America and Australia & Oceania (1.012***, 1.016**, and 1.027***) respectively (meaning that the incidence rate of both the new Covid-19 deaths are decreasing). The increase in COVID-19 instances owing to a rise in average temperature was also recorded in New York, United States, and Jakarta, Indonesia [3,31]. The temperature was also examined by Xie and Zhu [2] as a component of COVID-19 transmission. Wu et al. [20] reported that a 1 °C increase in temperature led to a 3.08% increase in new cases.

**Table 4:** Generalized Poisson regression analysis (DV: Total Cases (log)).

Variables	IRR						
	World	Asia	Europe	Africa	South America	North America	Australia & Oceania
**Temperature**	0.991***	0.996***	0.993***	0.978***	0.990***	1.032***	0.993***
	(0.0128)	(0.0254)	(0.00454)	(0.0570)	(0.00359)	(0.00106)	(0.00178)
**Precipitation**	0.993***	0.996***	0.998***	0.995***	0.993***	0.996***	0.993***
	(0.00147)	(0.00235)	(0.00713)	(0.00285)	(0.00541)	(0.009.61)	(0.0222)
**Population Density**	0.998***	1.006***	0.996***	0.997***	0.997***	1.045***	1.027***
	(0.00691)	(0.00154)	(0.00902)	(0.00157)	(0.00115)	(0.00191)	(0.00931)
**Days**	1.010***	1.016***	1.097***	1.010***	1.012***	1.093***	1.014***
	(0.00461)	(0.009.07)	(0.00878)	(0.0096)	(0.00124)	(0.00171)	(0.00277)
**Income Level**	1.098**	0.945***	1.053***	0.974***	1.138***	1.033***	1.607***
	(0.09082)	(0.01904)	(0.03342)	(0.02340)	(0.03882)	(0.08183)	(0.02830)
**_cons**	7.729***	8.668***	8.068***	11.404***	5.793***	4.958***	5.937***
	(0.248079)	(0.67513)	(0.777849	(0.15981)	(0.58719)	(0.85392)	(0.17388)
**Number of obs.**	134,723	30,567	33,770	34,091	22,398	8601	5296
**LR chi2 (df** = **5)**	61435***	15616***	15863***	13941***	12232***	6514***	3988***
**Pseudo R2**	0.83	0.33	0.57	0.47	0.73	0.13	0.15
**Goodness of fit tests**							
**Deviance**	129454.8	25645.57	28283.47	14619.18	27581.28*	7043.769	2819.756
**Pearson**	116022.8	22729.86	24097.2	12502.09	25990.77*	6351.62	2710.157

Notes: Where IRR represents the Incidence rate ratio. ***p < 0.01, **p < 0.05, *p < *0.1. Standard errors in parentheses*.

**Table 5:** Generalized Poisson regression analysis (DV: Total Deaths (log)).

Variables	IRR						
	World	Asia	Europe	Africa	South America	North America	Australia & Oceania
**Temperature**	0.982***	0.994***	1.012***	0.968***	0.948***	1.016**	1.027***
	(0.01685)	(0.03303)	(0.0583)	(0.07602)	(0.05101)	(0.13856)	(0.30172)
**Precipitation**	0.907***	0.996***	0.971***	0.989***	0.998**	0.963***	0.986***
	(0.00197)	0.00301)	(0.00917)	(0.003.89)	(0.00770)	(0.012347)	(0.003185)
**Population Density**	0.993***	0.994***	0.991***	0.912***	0.952***	1.042***	1.043***
	(0.00130)	(0.00261)	(0.00169)	(0.023132)	(0.0166712)	(0.023334)	(0.015141)
**Days**	1.106***	1.017***	1.095***	1.119**	1.116***	1.110***	1.178***
	(0.00628)	(0.00125)	(0.00115)	(0.00136)	(0.00175)	(0.00212)	(0.00466)
**Income Level**	1.273***	1.782***	1.057***	0.970***	1.274***	1.060***	2.659***
	(0.122020)	(0.25814)	(0.04123)	(0.33208)	(0.56351)	(0.95298)	(0.72799)
**_cons**	5.763***	5.106***	5.464***	9.039***	7.883***	6.368***	2.157***
	(0.02453)	(0.05192)	(0.06686)	(0.16512)	(0.11067)	(0.15640)	(0.10167)
**Number of obs.**	121,948	27,297	31,563	31,655	19,482	7806	4145
**LR chi2(5)**	52212***	9414***	12315***	13259***	20051***	5661***	3624***
**Pseudo R2**	0.82	0.678	0.56	0.51	0.79	0.13	0.19
**Goodness of fit tests**							
**Deviance**	158406	29853*	36062*	17457	32107***	5130	2881
**Pearson**	136346	24233	31012	14678	25580.63**	4502	2729

Notes: Where IRR represents the Incidence rate ratio. ***p < 0.01, **p < 0.05, *p < *0.1. Standard errors in parentheses*.

The precipitation also significantly influences the COVID-19 cases in the world. Findings suggest that the increase in daily precipitation negatively impacts the spread of COVID-19 in the world and all the continents except for Europe. For instance, in Table 4, the IRR for ‘precipitation’ indicates that for every 1 mm increase in daily precipitation, the predicted incidence rate ratio of COVID-19 cases decreased by 0.993 times in the world. The same is the case for all the continents and Covid-19 fatalities (Table 5). It suggests that the rise in precipitation was useful in halting the spread of COVID-19 worldwide. Wu et al. [20] reported that a 1% increase in relative humidity led to a 0.85% decrease in COVID-19 cases. Chan et al. [52] noted that a greater relative humidity reduces viral survival, confirming the empirical findings. Nevertheless, it is equally crucial that other climatic factors (air quality and wind speed) influence the spread of infectious illnesses [52].

Population density is positively associated with the spread of COVID-19 cases and fatalities in North America and Australia & Oceania while negatively associated with the spread of COVID-19 cases in Asia, Africa, Europe, and South America. Overall, the number of days that passed to the outbreak of the epidemic was positive while income level negatively impact the number of COVID-19 cases.

## 5 Conclusions

Global health and the economy face immense problems because of pathogens in history globally. The outbreak of the SARS-CoV-2 pathogen was responsible for the infectious disease of coronavirus (COVID-19). This research tried to assess the trend of COVID-19 cases, COVID-19 deaths, COVID-19 cases per million, and COVID-19 transmission rate in 202 affected countries, as of December 31, 2021. The 202 affected countries were divided into different panels according to their continent/geography, average annual temperature, average annual precipitation, population density, and per capita income. The day-wise median of confirmed COVID-19 new cases showed that the situation was different across time and continents. South America showed a higher median of daily COVID-19 cases on the 120th day (57), 150th day (272), 180th day (410), 200th day (631), 210th day (914), 240th day (933), 250th day (615), 270th day (696), 390th day (666), 400th day (949), 420th day (1204), 450th day (1877), 480th day (2162), 500th day (2518), 510th day (2517), 540th day (2148), and 550th day (1206) of the COVID-19 outbreak. Europe showed a higher median of daily COVID-19 cases on 90th day (63.5), 100th day (55.5), 300th day (920.5), 330th day (1377), 350th day (1416.5), 360th day (501.5), 650th day (935), 660th day (1311), 690th day (816), 700th day (1905), and 729th day (2012.5) of the COVID-19 outbreak. Asia showed a higher median of daily COVID-19 cases on 120th day (57), 570th day (995), 600th day (1366), and 630th day (1144) of the COVID-19 outbreak. Some possible factors behind the difference may be temperature, precipitation, population density, mobility, health sector, demographic indicators, etc. The trend analysis showed that the behavior of COVID-19 was different in different continents, temperature zones, precipitation levels, population density, and levels of income. Discussing the COVID-19 cases per million, the day-wise trend was comparatively higher for two groups of countries i.e., (a) average temperature below 7.5 °C and (b) average temperature between 7.5 °C and 15 °C, up to the 729th day of the outbreak. However, COVID-19 cases per million were comparatively low in the countries having an average temperature between 22.5 °C and 30 °C. Discussing the COVID-19 cases per million, the day-wise trend was comparatively higher for the countries having average precipitation between (a) 1 mm and 750 mm and (b) 750 mm and 1500 mm up to the 729th day of the outbreak. Discussing the COVID-19 cases per million, the day-wise trend was comparatively higher for the countries having more than 1000 people per sq. km. Discussing the COVID-19 cases per million, the day-wise trend was higher for the HICs, followed by UMICs, LMICs, and LIC. The confirmed COVID-19 cases were low in low-income countries, possibly due to less testing capability. It depicts that the virus-confirmed cases had a direct association with income per capita. To counter the spread of COVID-19, it is required to ensure awareness of the pandemic among the general population. Further, correlation analysis shows that COVID-19 new cases, total deaths, and transmission rate are significantly positively correlated with each other in the overall world and all the continents. Correlations coefficients indicate the negative and significant association between the temperature, precipitation, population density, income level, and COVID-19 cases, deaths, and transmission rate (TR) in the overall world. The temperature has a significant positive correlation with Covid-19 cases, deaths, and transmission rates in Europe and North America. The Poisson regression results also confirm this trend, the increase in temperature is significantly negatively related to both the Covid-19 cases and deaths in all the world and continents except North America. Similarly, daily precipitation negatively impacts the spread of COVID-19 in the world and all the continents except for Europe. Population density is also negatively related to COVID-19 cases and deaths in all the world and continents except North America and Australia & Oceania. However, Covid-19 cases and deaths increase with the number of days passed and the income level of the country.

## 6 Policy implementations and limitations

The findings of this study have important policy implications for governments and health authorities in managing COVID-19 or other pandemics in the future. Firstly, the study highlights the need for targeted interventions and responses based on the specific circumstances and factors affecting each country, including their geographical location, temperature, precipitation levels, population density, and per capita income. Secondly, the study emphasizes the importance of increased testing capacity, especially in low-income countries, to effectively control the spread of the virus. Thirdly, governments should prioritize awareness campaigns and education programs to increase public knowledge of the pandemic and its associated risks. The government should include the causes, symptoms, and precautions related to various viral diseases in the educational syllabus.

Several limitations of this study should be acknowledged. Firstly, the study provides evidence and results based on cross-sectional data, which has limitations in establishing causality. Secondly, there may be other factors that influence the spread of COVID-19 that were not included in this study. Thirdly, the study did not analyze the impact of government policies and interventions on the spread of the virus, which could be an important area for future research. Finally, this study did not consider the role of vaccination to reduce the spread of COVID-19. Vaccines are effective to combat and control the pandemic in the long run. The WHO has fixed 70% vaccination target for all countries and approved several COVID-19 vaccines.

## Author contribution statement

R. M. Ammar Zahid: Conceived and designed the experiments; Analyzed and interpreted the data; Wrote the paper.

Qamar Ali: Conceived and designed the experiments; Contributed reagents, materials, analysis tools or data; Wrote the paper.

Adil Saleem: Analyzed and interpreted the data; Contributed reagents, materials, analysis tools or data.

Judit Sági: Performed the experiments; Analyzed and interpreted the data.

## Data availability statement

Data will be made available on request.

## Declaration of competing interest

The authors declare that they have no known competing financial interests or personal relationships that could have appeared to influence the work reported in this paper.

## Figures and Tables

**Appendix 1:** List of COVID-19 affected countries (by continent)

Continent	COVID-19 affected countries
Asia (45 countries)	Afghanistan, Armenia, Azerbaijan, Bahrain, Bangladesh, Bhutan, Brunei Darussalam, Cambodia, China, Georgia, India, Indonesia, Iran, Iraq, Israel, Japan, Jordan, Kazakhstan, Kuwait, Kyrgyzstan, Laos, Lebanon, Malaysia, Maldives, Mongolia, Myanmar, Nepal, Oman, Pakistan, Palestine, Philippines, Qatar, Russia, Saudi Arabia, Singapore, South Korea, Sri Lanka, Syria, Thailand, Timor Leste, Turkey, United Arab Emirates, Uzbekistan, Vietnam, Yemen
Europe (50 countries)	Albania, Andorra, Austria, Belarus, Belgium, Bosnia and Herzegovina, Bulgaria, Croatia, Cyprus, Czechia, Denmark, Estonia, Faroe Islands, Finland, France, Germany, Gibraltar, Greece, Guernsey, Holy See, Hungary, Iceland, Ireland, Isle of Man, Italy, Jersey, Kosovo, Latvia, Liechtenstein, Lithuania, Luxembourg, Malta, Moldova, Monaco, Montenegro, Netherlands, North Macedonia, Norway, Poland, Portugal, Romania, San Marino, Serbia, Slovakia, Slovenia, Spain, Sweden, Switzerland, Ukraine, United Kingdom
Africa (52 countries)	Algeria, Angola, Benin, Botswana, Burkina Faso, Burundi, Cameroon, Cape Verde, Central African Republic, Chad, Congo, Cote d'Ivoire, Democratic Republic of the Congo, Djibouti, Egypt, Equatorial Guinea, Eritrea, Eswatini, Ethiopia, Gabon, Gambia, Ghana, Guinea, Guinea Bissau, Kenya, Liberia, Libya, Madagascar, Malawi, Mali, Mauritania, Mauritius, Morocco, Mozambique, Namibia, Niger, Nigeria, Rwanda, Sao Tome and Principe, Senegal, Seychelles, Sierra Leone, Somalia, South Africa, South Sudan, Sudan, Togo, Tunisia, Uganda, United Republic of Tanzania, Zambia, Zimbabwe
North America (34 countries)	Antigua and Barbuda, Aruba, Bahamas, Barbados, Belize, Bermuda, British Virgin Island, Canada, Cayman Islands, Costa Rica, Cuba, Curacao, Dominica, Dominican Republic, El Salvador, Greenland, Grenada, Guatemala, Haiti, Honduras, Jamaica, Mexico, Montserrat, Nicaragua, Panama, Puerto Rico, Saint Kitts and Nevis, Saint Lucia, Saint Vincent and the Grenadines, Sint Maarten, Trinidad and Tobago, Turks and Caicos island, United States of America, United States Virgin Islands
South America (13 countries)	Argentina, Bolivia, Brazil, Chile, Colombia, Ecuador, Falkland Islands, Guyana, Paraguay, Peru, Suriname, Uruguay, Venezuela
Australia and Oceania (8 countries)	Australia, Fiji, French Polynesia, Guam, New Caledonia, New Zealand, Northern Mariana Islands, Papua New Guinea

**Appendix 2:** List of COVID-19 affected countries (by average annual temperature)

Average annual temperature	COVID-19 affected countries
**Less than 7.50°C** (22 Countries)	Andorra, Armenia, Austria, Belarus, Canada, Czechia, Estonia, Falkland Islands, Faroe Islands, Finland, Greenland, Iceland, Kazakhstan, Latvia, Lithuania, Mongolia, Norway, Poland, Russia, Slovakia, Sweden, Switzerland
**7.50°C -15.00°C** (41 Countries)	Afghanistan, Albania, Azerbaijan, Belgium, Bosnia and Herzegovina, Bulgaria, Chile, China, Croatia, Denmark, France, Georgia, Germany, Guernsey, Holy See, Hungary, Ireland, Isle of Man, Italy, Japan, Jersey, Kosovo, Kyrgyzstan, Liechtenstein, Luxembourg, Moldova, Monaco, Montenegro, Netherlands, New Zealand, North Macedonia, Romania, San Marino, Serbia, Slovenia, South Korea, Turkey, Ukraine, United Kingdom, United States of America, Uzbekistan
**15.01°C-22.00°C** (46 Countries)	Algeria, Angola, Argentina, Australia, Bermuda, Bhutan, Bolivia, Botswana, Burundi, Cyprus, Ecuador, Egypt, Eswatini, Ethiopia, Gibraltar, Greece, Guatemala, Iran, Iraq, Israel, Jordan, Kenya, Lebanon, Libya, Madagascar, Malawi, Malta, Mexico, Morocco, Namibia, Nepal, Pakistan, Palestine, Paraguay, Peru, Portugal, Rwanda, South Africa, Spain, Syria, Tunisia, Uganda, United Republic of Tanzania, Uruguay, Zambia, Zimbabwe
**22.01°C-30.00°C** (93 Countries)	Antigua and Barbuda, Aruba, Bahamas, Bahrain, Bangladesh, Barbados, Belize, Benin, Brazil, British Virgin Island, Brunei Darussalam, Burkina Faso, Cambodia, Cameroon, Cape Verde, Cayman Islands, Central African Republic, Chad, Colombia, Congo, Costa Rica, Cote d’voire, Cuba, Curacao, Democratic Republic of the Congo, Djibouti, Dominica, Dominican Republic, El Salvador, Equatorial Guinea, Eritrea, Fiji, French Polynesia, Gabon, Gambia, Ghana, Grenada, Guam, Guinea, Guinea Bissau, Guyana, Haiti, Honduras, India, Indonesia, Jamaica, Kuwait, Laos, Liberia, Malaysia, Maldives, Mali, Mauritania, Mauritius, Montserrat, Mozambique, Myanmar, New Caledonia, Nicaragua, Niger, Nigeria, Northern Mariana Islands, Oman, Panama, Papua New Guinea, Philippines, Puerto Rico, Qatar, Saint Kitts and Nevis, Saint Lucia, Saint Vincent and the Grenadines, Sao Tome and Principe, Saudi Arabia, Senegal, Seychelles, Sierra Leone, Singapore, Sint Maarten, Somalia, South Sudan, Sri Lanka, Sudan, Suriname, Thailand, Timor Leste, Togo, Trinidad and Tobago, Turks and Caicos Island, United Arab Emirates, United States Virgin Islands, Venezuela, Vietnam, Yemen

**Appendix 3:** List of COVID-19 affected countries (by average annual precipitation)

Average annual precipitation	COVID-19 affected countries
**1 mm-750 mm** (75 Countries)	Afghanistan, Algeria, Antigua and Barbuda, Armenia, Aruba, Azerbaijan, Bahrain, Belarus, Botswana, Bulgaria, Cape Verde, Chad, Cote d'Ivoire, Curacao, Cyprus, Czechia, Denmark, Djibouti, Egypt, Eritrea, Estonia, Falkland Islands, Finland, Germany, Greece, Greenland, Guernsey, Hungary, Iran, Iraq, Israel, Jersey, Jordan, Kazakhstan, Kosovo, Kuwait, Kyrgyzstan, Latvia, Libya, Lithuania, Mali, Malta, Mauritania, Moldova, Mongolia, Morocco, Namibia, Niger, North Macedonia, Oman, Pakistan, Palestine, Peru, Poland, Qatar, Romania, Russia, Saudi Arabia, Senegal, Serbia, Sint Maarten, Slovakia, Somalia, South Africa, Spain, Sudan, Sweden, Syria, Tunisia, Turkey, Turks and Caicos Island, Ukraine, United Arab Emirates, Uzbekistan, Yemen
**751 mm-1500 mm** (75 Countries)	Albania, Andorra, Angola, Argentina, Australia, Austria, Bahamas, Barbados, Belgium, Benin, Bhutan, Bolivia, Bosnia and Herzegovina, Brazil, British Virgin Island, Burkina Faso, Burundi, Canada, Cayman Islands, Central African Republic, Chile, China, Croatia, Cuba, Dominican Republic, Ecuador, Eswatini, Ethiopia, Faroe Islands, France, Gambia, Georgia, Ghana, Gibraltar, Holy See, Iceland, India, Ireland, Isle of Man, Italy, Jamaica, Kenya, Lebanon, Liechtenstein, Luxembourg, Malawi, Mexico, Monaco, Montenegro, Mozambique, Netherlands, New Caledonia, New Zealand, Nigeria, Norway, Portugal, Rwanda, Saint Kitts and Nevis, San Marino, Slovenia, South Korea, South Sudan, Switzerland, Timor Leste, Togo, Trinidad and Tobago, Uganda, United Kingdom, United Republic of Tanzania, United States of America, United States Virgin Islands, Uruguay, Venezuela, Zambia, Zimbabwe
**1501 mm-2250 mm** (37 Countries)	Belize, Bermuda, Cambodia, Cameroon, Colombia, Congo, Costa Rica, Democratic Republic of the Congo, Dominica, El Salvador, French Polynesia, Gabon, Grenada, Guatemala, Guinea, Guinea Bissau, Haiti, Honduras, Japan, Laos, Madagascar, Maldives, Mauritius, Montserrat, Nepal, Northern Mariana Islands, Panama, Paraguay, Puerto Rico, Saint Lucia, Saint Vincent and the Grenadines, Sao Tome and Principe, Singapore, Sri Lanka, Suriname, Thailand, Vietnam
**2251 mm-3000 mm** (15 Countries)	Bangladesh, Brunei Darussalam, Equatorial Guinea, Fiji, Guam, Guyana, Indonesia, Liberia, Malaysia, Myanmar, Nicaragua, Papua New Guinea, Philippines, Seychelles, Sierra Leone

**Appendix 4:** List of COVID-19 affected countries (by population density)

Populating Density	COVID-19 affected countries
**Less than 150 people per sq. km** (138 Countries)	Afghanistan, Albania, Algeria, Angola, Argentina, Armenia, Australia, Austria, Azerbaijan, Bahamas, Belarus, Belize, Benin, Bhutan, Bolivia, Bosnia and Herzegovina, Botswana, Brazil, Brunei Darussalam, Bulgaria, Burkina Faso, Cambodia, Cameroon, Canada, Cape Verde, Central African Republic, Chad, Chile, China, Colombia, Congo, Costa Rica, Cote d’voire, Croatia, Cuba, Cyprus, Czechia, Democratic Republic of the Congo, Denmark, Djibouti, Dominica, Ecuador, Egypt, Equatorial Guinea, Eritrea, Estonia, Eswatini, Ethiopia, Falkland Islands, Faroe Islands, Fiji, Finland, France, French Polynesia, Gabon, Georgia, Ghana, Greece, Greenland, Guinea, Guinea Bissau, Guyana, Honduras, Hungary, Iceland, Indonesia, Iran, Iraq, Ireland, Isle of Man, Jordan, Kazakhstan, Kenya, Kyrgyzstan, Laos, Latvia, Liberia, Libya, Lithuania, Madagascar, Malaysia, Mali, Mauritania, Mexico, Moldova, Mongolia, Montenegro, Montserrat, Morocco, Mozambique, Myanmar, Namibia, New Caledonia, New Zealand, Nicaragua, Niger, North Macedonia, Northern Mariana Islands, Norway, Oman, Panama, Papua New Guinea, Paraguay, Peru, Poland, Portugal, Romania, Russia, Saudi Arabia, Senegal, Serbia, Sierra Leone, Slovakia, Slovenia, Somalia, South Africa, South Sudan, Spain, Sudan, Suriname, Sweden, Syria, Thailand, Timor Leste, Togo, Tunisia, Turkey, Turks and Caicos Island, Ukraine, United Arab Emirates, United Republic of Tanzania, United States of America, Uruguay, Uzbekistan, Venezuela, Yemen, Zambia, Zimbabwe
**151**–**300 people per sq. km** (28 Countries)	Andorra, Antigua & Barbuda, British Virgin Island, Cayman Islands, Dominican Republic, Gambia, Germany, Guatemala, Italy, Jamaica, Kosovo, Kuwait, Liechtenstein, Luxembourg, Malawi, Nepal, Nigeria, Pakistan, Qatar, Saint Kitts and Nevis, Saint Lucia, Saint Vincent and the Grenadines, Sao Tome and Principe, Seychelles, Switzerland, Trinidad and Tobago, Uganda, United Kingdom,
**301**–**450 people per sq. km** (14 Countries)	Belgium, Burundi, Curacao, El Salvador, Grenada, Guam, Haiti, Israel, Japan, Philippines, Puerto Rico, Sri Lanka, United States Virgin Islands, Vietnam
**451**–**600 people per sq. km** (06 Countries)	Aruba, India, Netherlands, Rwanda, San Marino, South Korea
**601**–**1000 people per sq. km** (07 Countries)	Barbados, Guernsey, Jersey, Lebanon, Mauritius, Palestine, Sint Maarten,
**Above 1000 people per sq. km** (09 Countries)	Bahrain, Bangladesh, Bermuda, Gibraltar, Holy See, Maldives, Malta, Monaco, Singapore

**Appendix 5:** List of COVID-19 affected countries (by income level)

Income Level	COVID-19 affected countries
**High-Income Countries** (79 Countries)	Andorra, Antigua and Barbuda, Aruba, Australia, Austria, Bahamas, Bahrain, Barbados, Belgium, Bermuda, British Virgin Island, Brunei Darussalam, Canada, Cayman Islands, Chile, Croatia, Curacao, Cyprus, Czechia, Denmark, Estonia, Falkland Islands, Faroe Islands, Finland, France, French Polynesia, Germany, Gibraltar, Greece, Greenland, Guam, Guernsey, Holy See, Hungary, Iceland, Ireland, Isle of Man, Israel, Italy, Japan, Jersey, Kuwait, Latvia, Liechtenstein, Lithuania, Luxembourg, Malta, Monaco, Montserrat, Netherlands, New Caledonia, New Zealand, Northern Mariana Islands, Norway, Oman, Panama, Poland, Portugal, Puerto Rico, Qatar, Saint Kitts and Nevis, San Marino, Saudi Arabia, Seychelles, Singapore, Sint Maarten, Slovakia, Slovenia, South Korea, Spain, Sweden, Switzerland, Trinidad and Tobago, Turks and Caicos Island, United Arab Emirates, United Kingdom, United States of America, United States Virgin Islands, Uruguay
**Upper Middle Income Countries** (55 Countries)	Albania, Algeria, Argentina, Armenia, Azerbaijan, Belarus, Belize, Bosnia and Herzegovina, Botswana, Brazil, Bulgaria, China, Colombia, Costa Rica, Cuba, Dominica, Dominican Republic, Ecuador, Equatorial Guinea, Fiji, Gabon, Georgia, Grenada, Guatemala, Guyana, Iran, Iraq, Jamaica, Jordan, Kazakhstan, Kosovo, Lebanon, Libya, Malaysia, Maldives, Mauritius, Mexico, Montenegro, Namibia, North Macedonia, Paraguay, Peru, Romania, Russia, Saint Lucia, Saint Vincent and the Grenadines, Serbia, South Africa, Sri Lanka, Suriname, Thailand, Turkey, Venezuela,
**Lower Middle Income Countries** (40 Countries)	Angola, Bangladesh, Bhutan, Bolivia, Cambodia, Cameroon, Cape Verde, Cote d'Ivoire, Djibouti, Egypt, El Salvador, Eswatini, Ghana, Honduras, India, Indonesia, Kenya, Kyrgyzstan, Laos, Mauritania, Moldova, Mongolia, Morocco, Myanmar, Nicaragua, Niger, Pakistan, Palestine, Papua New Guinea, Philippines, Sao Tome and Principe, Senegal, Sudan, Timor Leste, Tunisia, Ukraine, Uzbekistan, Vietnam, Zambia, Zimbabwe
**Low-Income Countries** (30 Countries)	Afghanistan, Benin, Burkina Faso, Burundi, Central African Republic, Chad, Congo, Democratic Republic of the Congo, Eritrea, Ethiopia, Gambia, Guinea, Guinea Bissau, Haiti, Liberia, Madagascar, Malawi, Mali, Mozambique, Nepal, Nigeria, Rwanda, Sierra Leone, Somalia, South Sudan, Syria, Togo, Uganda, United Republic of Tanzania, Yemen
